# 3-{[3-(4-Chloro­phen­yl)-4,5-dihydro-1,2-oxazol-5-yl]meth­yl}-1,5-dimethyl-1*H*-1,5-benzodiazepine-2,4(3*H*,5*H*)-dione

**DOI:** 10.1107/S160053681100657X

**Published:** 2011-02-26

**Authors:** Rachida Dardouri, Youssef Kandri Rodi, Sonia Ladeira, El Mokhtar Essassi, Seik Weng Ng

**Affiliations:** a Laboratoire de Chimie Organique Appliquée, Faculté des Sciences et Techniques Université Sidi Mohamed Ben Abdallah, Fés, Morocco; b Service Commun Rayons-X FR2599, Université Paul Sabatier, Bâtiment 2R1, 118 route de Narbonne, Toulouse, France; c Laboratoire de Chimie Organique Hétérocyclique, Pôle de Compétences Pharmacochimie, Université Mohammed V-Agdal, BP 1014 Avenue Ibn Batout, Rabat, Morocco; d Department of Chemistry, University of Malaya, 50603 Kuala Lumpur, Malaysia

## Abstract

The seven-membered ring of the title mol­ecule, C_21_H_20_ClN_3_O_3_, adopts a boat-shaped conformation (with the C atoms of the fused-ring as the stern and the methine C atom as the prow). The substituent at the 3-position occupies an equatorial position; its five-membered ring is approximately planar (r.m.s. deviation = 0.081 Å), and is aligned at 14.5 (1)° with respect to the chloro­phenyl ring to which it is connected.

## Related literature

For the crystal structure of the tetra­decyl-substituted analog, see: Dardouri *et al.* (2011 ▶).
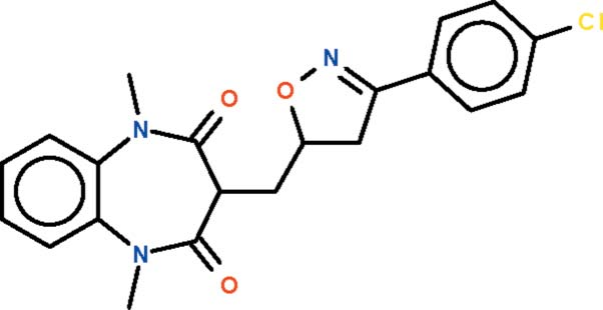


## Experimental

### 

#### Crystal data

C_21_H_20_ClN_3_O_3_
*M* *_r_* = 397.85
Triclinic, 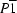

*a* = 8.1821 (1) Å
*b* = 9.0741 (1) Å
*c* = 13.3792 (2) Å
α = 79.748 (1)°
β = 80.142 (1)°
γ = 85.910 (1)°
*V* = 962.19 (2) Å^3^
*Z* = 2
Mo *K*α radiation
μ = 0.23 mm^−1^
*T* = 295 K
0.40 × 0.30 × 0.20 mm

#### Data collection

Bruker X8 APEXII diffractometer
Absorption correction: multi-scan (*SADABS*; Sheldrick, 1996 ▶) *T*_min_ = 0.915, *T*_max_ = 0.956
19243 measured reflections
4383 independent reflections
4056 reflections with *I* > 2σ(*I*)
*R*_int_ = 0.020

#### Refinement

*R*[*F* ^2^ > 2σ(*F* ^2^)] = 0.066
*wR*(*F* ^2^) = 0.184
*S* = 1.03
4383 reflections
256 parameters
H-atom parameters constrained
Δρ_max_ = 0.83 e Å^−3^
Δρ_min_ = −0.44 e Å^−3^

### 

Data collection: *APEX2* (Bruker, 2008 ▶); cell refinement: *SAINT* (Bruker, 2008 ▶); data reduction: *SAINT*; program(s) used to solve structure: *SHELXS97* (Sheldrick, 2008 ▶); program(s) used to refine structure: *SHELXL97* (Sheldrick, 2008 ▶); molecular graphics: *X-SEED* (Barbour, 2001 ▶); software used to prepare material for publication: *publCIF* (Westrip, 2010 ▶).

## Supplementary Material

Crystal structure: contains datablocks global, I. DOI: 10.1107/S160053681100657X/bt5479sup1.cif

Structure factors: contains datablocks I. DOI: 10.1107/S160053681100657X/bt5479Isup2.hkl

Additional supplementary materials:  crystallographic information; 3D view; checkCIF report

## Figures and Tables

**Fig. 1.**
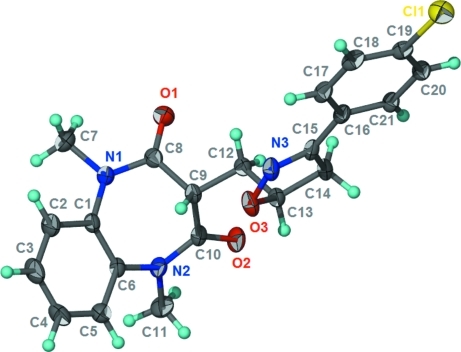
Anisotropic displacement ellipsoid plot (Barbour, 2001) of C21H20ClN3O3 at the 50% probability level; hydrogen atoms are drawn as arbitrary radius.

## supplementary crystallographic information

### Comment

The methylene part of 1,5-dimethyl-1,5-benzodiazepine-2,4-dione is relatively acidic, and one proton can be abstracted by using potassium *t*-butoxide; the resulting carbanion can undergo a nucleophlilic subsitution with a dibromoalkane to form 3-substituted derivatives. In a previous study, the compound was reacted with bromotetradecane to give the tetradecyl substitued derivative (Dardouri *et al.*, 2011). The title compound was obtained by using *p*-chlorobenzaldoxime to react with the ally group to furnish the title isoxazolinyl derivative (Scheme I, Fig. 1). The seven-membered ring of C_21_H_20_ClN_3_O_3_ adopts a boat-shaped conformation (with the C atoms of the fused-ring as the stern and the methine C atom as the prow). The substituent at the 3-position occupies an equatorial position.

### Experimental

To a solution of 3-allyl-1,5-dimethyl-1,5-benzodiazepine-2,4-dione (0.25 g, 1 mmol) and *p*-chlorobenzaldoxime (0.2 g, 1.3 mmol) in chloroform (10 ml) was added to a 4%solution of sodium hypochlorite solution (commerical bleach) (4 ml) at 273 K. Stirring was continued for 4 h. The organic layer was dried and the solvent evaporated under reduced pressure. The residue was then purified by column chromatography on silica gel by using a mixture of hexane and ethyl acetate (1/1) as eluent. Colorless crystals were isolated when the solvent was allowed to evaporate.

### Refinement

H-atoms were placed in calculated positions (C—H 0.93–0.97 Å) and were included in the refinement in the riding model approximation, with *U*(H) set to 1.2–1.5*U*_eq_(C).

Omitted from the refinements were the following reflections because of being obscured by  the beam stop and/or bad agreement between observed and calculated structure factors: (0 1 0), (1 1 0), (0 2 0), (2 3 0), (0 0 1), (1 1 1), (-1 1 2), (0 1 2) and (1 1 2).

### Crystal data

**Table:**

C_21_H_20_ClN_3_O_3_	*Z* = 2
*M**_r_* = 397.85	*F*(000) = 416
Triclinic, *P*1	*D*_x_ = 1.373 Mg m^−^^3^
Hall symbol: -P 1	Mo *K*α radiation, λ = 0.71073 Å
*a* = 8.1821 (1) Å	Cell parameters from 9887 reflections
*b* = 9.0741 (1) Å	θ = 3.0–30.7°
*c* = 13.3792 (2) Å	µ = 0.23 mm^−^^1^
α = 79.748 (1)°	*T* = 295 K
β = 80.142 (1)°	Block, colorless
γ = 85.910 (1)°	0.40 × 0.30 × 0.20 mm
*V* = 962.19 (2) Å^3^	

### Data collection

**Table:**

Bruker X8 APEXII diffractometer	4383 independent reflections
Radiation source: fine-focus sealed tube	4056 reflections with *I* > 2σ(*I*)
graphite	*R*_int_ = 0.020
φ and ω scans	θ_max_ = 27.5°, θ_min_ = 3.0°
Absorption correction: multi-scan (*SADABS*; Sheldrick, 1996)	*h* = −10→10
*T*_min_ = 0.915, *T*_max_ = 0.956	*k* = −11→11
19243 measured reflections	*l* = −17→17

### Refinement

**Table:**

Refinement on *F*^2^	Secondary atom site location: difference Fourier map
Least-squares matrix: full	Hydrogen site location: inferred from neighbouring sites
*R*[*F*^2^ > 2σ(*F*^2^)] = 0.066	H-atom parameters constrained
*wR*(*F*^2^) = 0.184	*w* = 1/[σ^2^(*F*_o_^2^) + (0.1062*P*)^2^ + 0.889*P*] where *P* = (*F*_o_^2^ + 2*F*_c_^2^)/3
*S* = 1.03	(Δ/σ)_max_ = 0.001
4383 reflections	Δρ_max_ = 0.83 e Å^−^^3^
256 parameters	Δρ_min_ = −0.44 e Å^−^^3^
0 restraints	Extinction correction: *SHELXL97* (Sheldrick, 2008), Fc^*^=kFc[1+0.001xFc^2^λ^3^/sin(2θ)]^-1/4^
Primary atom site location: structure-invariant direct methods	Extinction coefficient: 0.046 (8)

### Fractional atomic coordinates and isotropic or equivalent isotropic displacement parameters (Å^2^)

**Table:**

	*x*	*y*	*z*	*U*_iso_*/*U*_eq_	
Cl1	0.00333 (8)	0.14321 (7)	1.07254 (5)	0.0471 (2)	
O1	0.2880 (2)	1.2329 (2)	0.66476 (13)	0.0428 (4)	
O2	0.6469 (2)	1.2468 (2)	0.82002 (12)	0.0430 (4)	
O3	0.6108 (2)	0.79540 (18)	0.76659 (12)	0.0400 (4)	
N1	0.5122 (2)	1.2697 (2)	0.53908 (14)	0.0337 (4)	
N2	0.7763 (2)	1.2907 (2)	0.65440 (13)	0.0310 (4)	
N3	0.5112 (2)	0.6671 (2)	0.78978 (14)	0.0353 (4)	
C1	0.6816 (3)	1.2379 (2)	0.50002 (15)	0.0309 (4)	
C2	0.7216 (3)	1.2001 (3)	0.40171 (18)	0.0452 (6)	
H2	0.6374	1.1910	0.3647	0.054*	
C3	0.8856 (4)	1.1760 (4)	0.35894 (19)	0.0504 (7)	
H3	0.9109	1.1522	0.2931	0.060*	
C4	1.0116 (3)	1.1870 (3)	0.4132 (2)	0.0448 (6)	
H4	1.1216	1.1710	0.3839	0.054*	
C5	0.9744 (3)	1.2219 (3)	0.51136 (18)	0.0360 (5)	
H5	1.0596	1.2275	0.5483	0.043*	
C6	0.8100 (3)	1.2487 (2)	0.55536 (15)	0.0284 (4)	
C7	0.4069 (3)	1.3563 (3)	0.46807 (19)	0.0449 (6)	
H7A	0.3220	1.4127	0.5059	0.067*	
H7B	0.3563	1.2889	0.4359	0.067*	
H7C	0.4739	1.4237	0.4163	0.067*	
C8	0.4365 (3)	1.2097 (2)	0.63486 (16)	0.0313 (4)	
C9	0.5486 (3)	1.1145 (2)	0.70220 (15)	0.0293 (4)	
H9	0.6178	1.0465	0.6616	0.035*	
C10	0.6618 (3)	1.2213 (2)	0.73248 (15)	0.0301 (4)	
C11	0.8826 (3)	1.3976 (3)	0.6799 (2)	0.0437 (6)	
H11A	0.8160	1.4626	0.7216	0.065*	
H11B	0.9365	1.4563	0.6177	0.065*	
H11C	0.9648	1.3438	0.7173	0.065*	
C12	0.4489 (3)	1.0212 (2)	0.79575 (16)	0.0307 (4)	
H12A	0.4059	1.0846	0.8463	0.037*	
H12B	0.3551	0.9814	0.7752	0.037*	
C13	0.5550 (3)	0.8928 (2)	0.84372 (16)	0.0326 (4)	
H13	0.6499	0.9304	0.8659	0.039*	
C14	0.4547 (3)	0.7892 (2)	0.93283 (15)	0.0317 (4)	
H14A	0.5181	0.7544	0.9881	0.038*	
H14B	0.3515	0.8382	0.9599	0.038*	
C15	0.4243 (3)	0.6634 (2)	0.87944 (15)	0.0301 (4)	
C16	0.3142 (2)	0.5391 (2)	0.92464 (15)	0.0290 (4)	
C17	0.2745 (3)	0.4381 (3)	0.86525 (16)	0.0352 (5)	
H17	0.3144	0.4520	0.7950	0.042*	
C18	0.1772 (3)	0.3186 (3)	0.90967 (18)	0.0367 (5)	
H18	0.1508	0.2523	0.8698	0.044*	
C19	0.1190 (3)	0.2982 (2)	1.01501 (17)	0.0330 (4)	
C20	0.1526 (3)	0.3979 (2)	1.07529 (16)	0.0310 (4)	
H20	0.1109	0.3840	1.1453	0.037*	
C21	0.2496 (3)	0.5188 (2)	1.02965 (15)	0.0291 (4)	
H21	0.2718	0.5871	1.0693	0.035*	

### Atomic displacement parameters (Å^2^)

**Table:**

	*U*^11^	*U*^22^	*U*^33^	*U*^12^	*U*^13^	*U*^23^
Cl1	0.0506 (4)	0.0392 (3)	0.0554 (4)	−0.0106 (2)	−0.0094 (3)	−0.0145 (3)
O1	0.0328 (8)	0.0558 (11)	0.0374 (9)	0.0037 (7)	−0.0019 (6)	−0.0071 (7)
O2	0.0561 (10)	0.0510 (10)	0.0238 (7)	−0.0044 (8)	−0.0058 (7)	−0.0114 (7)
O3	0.0490 (9)	0.0360 (8)	0.0274 (7)	0.0054 (7)	0.0083 (6)	−0.0011 (6)
N1	0.0331 (9)	0.0421 (10)	0.0258 (8)	−0.0033 (7)	−0.0058 (7)	−0.0037 (7)
N2	0.0381 (9)	0.0322 (9)	0.0248 (8)	−0.0041 (7)	−0.0079 (7)	−0.0063 (7)
N3	0.0432 (10)	0.0348 (9)	0.0241 (8)	0.0085 (8)	−0.0014 (7)	−0.0026 (7)
C1	0.0339 (10)	0.0360 (10)	0.0220 (9)	−0.0061 (8)	−0.0025 (7)	−0.0030 (7)
C2	0.0481 (13)	0.0640 (16)	0.0256 (10)	−0.0096 (11)	−0.0045 (9)	−0.0121 (10)
C3	0.0542 (15)	0.0698 (18)	0.0267 (11)	−0.0104 (13)	0.0074 (10)	−0.0166 (11)
C4	0.0384 (12)	0.0523 (14)	0.0400 (12)	−0.0074 (10)	0.0093 (10)	−0.0104 (11)
C5	0.0338 (11)	0.0379 (11)	0.0356 (11)	−0.0038 (8)	−0.0045 (8)	−0.0042 (9)
C6	0.0346 (10)	0.0284 (9)	0.0214 (9)	−0.0045 (7)	−0.0037 (7)	−0.0018 (7)
C7	0.0408 (12)	0.0581 (15)	0.0366 (12)	−0.0014 (11)	−0.0149 (10)	−0.0020 (11)
C8	0.0338 (10)	0.0339 (10)	0.0271 (10)	−0.0031 (8)	−0.0027 (8)	−0.0091 (8)
C9	0.0326 (10)	0.0301 (9)	0.0235 (9)	−0.0002 (7)	−0.0005 (7)	−0.0045 (7)
C10	0.0370 (10)	0.0306 (10)	0.0224 (9)	0.0026 (8)	−0.0059 (7)	−0.0042 (7)
C11	0.0472 (13)	0.0469 (13)	0.0433 (13)	−0.0099 (10)	−0.0154 (10)	−0.0137 (10)
C12	0.0292 (9)	0.0346 (10)	0.0267 (9)	−0.0003 (8)	−0.0023 (7)	−0.0030 (8)
C13	0.0309 (10)	0.0381 (11)	0.0263 (9)	0.0005 (8)	−0.0025 (7)	−0.0016 (8)
C14	0.0391 (11)	0.0322 (10)	0.0211 (9)	−0.0001 (8)	−0.0008 (8)	−0.0015 (7)
C15	0.0358 (10)	0.0327 (10)	0.0202 (8)	0.0085 (8)	−0.0057 (7)	−0.0033 (7)
C16	0.0311 (10)	0.0328 (10)	0.0237 (9)	0.0069 (8)	−0.0067 (7)	−0.0075 (7)
C17	0.0383 (11)	0.0450 (12)	0.0250 (9)	0.0090 (9)	−0.0086 (8)	−0.0145 (8)
C18	0.0383 (11)	0.0415 (12)	0.0374 (11)	0.0085 (9)	−0.0143 (9)	−0.0216 (9)
C19	0.0306 (10)	0.0337 (10)	0.0376 (11)	0.0033 (8)	−0.0095 (8)	−0.0116 (8)
C20	0.0337 (10)	0.0336 (10)	0.0267 (9)	0.0002 (8)	−0.0044 (8)	−0.0094 (8)
C21	0.0349 (10)	0.0304 (10)	0.0231 (9)	0.0029 (8)	−0.0056 (7)	−0.0085 (7)

### Geometric parameters (Å, °)

**Table:**

Cl1—C19	1.738 (2)	C9—C12	1.523 (3)
O1—C8	1.228 (3)	C9—C10	1.536 (3)
O2—C10	1.218 (3)	C9—H9	0.9800
O3—N3	1.427 (3)	C11—H11A	0.9600
O3—C13	1.470 (3)	C11—H11B	0.9600
N1—C8	1.360 (3)	C11—H11C	0.9600
N1—C1	1.423 (3)	C12—C13	1.518 (3)
N1—C7	1.477 (3)	C12—H12A	0.9700
N2—C10	1.371 (3)	C12—H12B	0.9700
N2—C6	1.420 (3)	C13—C14	1.534 (3)
N2—C11	1.467 (3)	C13—H13	0.9800
N3—C15	1.282 (3)	C14—C15	1.506 (3)
C1—C2	1.397 (3)	C14—H14A	0.9700
C1—C6	1.403 (3)	C14—H14B	0.9700
C2—C3	1.384 (4)	C15—C16	1.471 (3)
C2—H2	0.9300	C16—C21	1.398 (3)
C3—C4	1.378 (4)	C16—C17	1.403 (3)
C3—H3	0.9300	C17—C18	1.376 (4)
C4—C5	1.383 (3)	C17—H17	0.9300
C4—H4	0.9300	C18—C19	1.392 (3)
C5—C6	1.396 (3)	C18—H18	0.9300
C5—H5	0.9300	C19—C20	1.384 (3)
C7—H7A	0.9600	C20—C21	1.388 (3)
C7—H7B	0.9600	C20—H20	0.9300
C7—H7C	0.9600	C21—H21	0.9300
C8—C9	1.514 (3)		
			
N3—O3—C13	109.02 (15)	N2—C11—H11B	109.5
C8—N1—C1	123.46 (18)	H11A—C11—H11B	109.5
C8—N1—C7	117.58 (19)	N2—C11—H11C	109.5
C1—N1—C7	118.47 (18)	H11A—C11—H11C	109.5
C10—N2—C6	122.62 (17)	H11B—C11—H11C	109.5
C10—N2—C11	117.51 (18)	C13—C12—C9	111.22 (17)
C6—N2—C11	119.31 (18)	C13—C12—H12A	109.4
C15—N3—O3	109.03 (18)	C9—C12—H12A	109.4
C2—C1—C6	119.0 (2)	C13—C12—H12B	109.4
C2—C1—N1	118.8 (2)	C9—C12—H12B	109.4
C6—C1—N1	122.17 (18)	H12A—C12—H12B	108.0
C3—C2—C1	120.5 (2)	O3—C13—C12	107.78 (17)
C3—C2—H2	119.8	O3—C13—C14	103.57 (17)
C1—C2—H2	119.8	C12—C13—C14	112.48 (17)
C4—C3—C2	120.5 (2)	O3—C13—H13	110.9
C4—C3—H3	119.8	C12—C13—H13	110.9
C2—C3—H3	119.8	C14—C13—H13	110.9
C3—C4—C5	119.9 (2)	C15—C14—C13	100.88 (16)
C3—C4—H4	120.0	C15—C14—H14A	111.6
C5—C4—H4	120.0	C13—C14—H14A	111.6
C4—C5—C6	120.5 (2)	C15—C14—H14B	111.6
C4—C5—H5	119.7	C13—C14—H14B	111.6
C6—C5—H5	119.7	H14A—C14—H14B	109.4
C5—C6—C1	119.58 (19)	N3—C15—C16	120.6 (2)
C5—C6—N2	119.19 (19)	N3—C15—C14	114.1 (2)
C1—C6—N2	121.20 (18)	C16—C15—C14	125.22 (17)
N1—C7—H7A	109.5	C21—C16—C17	118.9 (2)
N1—C7—H7B	109.5	C21—C16—C15	119.55 (19)
H7A—C7—H7B	109.5	C17—C16—C15	121.59 (19)
N1—C7—H7C	109.5	C18—C17—C16	120.8 (2)
H7A—C7—H7C	109.5	C18—C17—H17	119.6
H7B—C7—H7C	109.5	C16—C17—H17	119.6
O1—C8—N1	122.1 (2)	C17—C18—C19	119.1 (2)
O1—C8—C9	122.53 (19)	C17—C18—H18	120.4
N1—C8—C9	115.32 (18)	C19—C18—H18	120.4
C8—C9—C12	111.58 (17)	C20—C19—C18	121.4 (2)
C8—C9—C10	107.09 (17)	C20—C19—Cl1	119.19 (17)
C12—C9—C10	112.23 (17)	C18—C19—Cl1	119.38 (17)
C8—C9—H9	108.6	C21—C20—C19	119.01 (19)
C12—C9—H9	108.6	C21—C20—H20	120.5
C10—C9—H9	108.6	C19—C20—H20	120.5
O2—C10—N2	122.0 (2)	C20—C21—C16	120.69 (19)
O2—C10—C9	121.92 (19)	C20—C21—H21	119.7
N2—C10—C9	116.09 (17)	C16—C21—H21	119.7
N2—C11—H11A	109.5		
			
C13—O3—N3—C15	−11.1 (2)	C11—N2—C10—C9	177.93 (19)
C8—N1—C1—C2	132.2 (2)	C8—C9—C10—O2	109.6 (2)
C7—N1—C1—C2	−39.5 (3)	C12—C9—C10—O2	−13.1 (3)
C8—N1—C1—C6	−50.2 (3)	C8—C9—C10—N2	−68.0 (2)
C7—N1—C1—C6	138.0 (2)	C12—C9—C10—N2	169.27 (17)
C6—C1—C2—C3	−1.0 (4)	C8—C9—C12—C13	162.03 (18)
N1—C1—C2—C3	176.6 (2)	C10—C9—C12—C13	−77.8 (2)
C1—C2—C3—C4	0.9 (4)	N3—O3—C13—C12	−101.45 (18)
C2—C3—C4—C5	0.2 (4)	N3—O3—C13—C14	17.9 (2)
C3—C4—C5—C6	−1.1 (4)	C9—C12—C13—O3	−61.8 (2)
C4—C5—C6—C1	1.0 (3)	C9—C12—C13—C14	−175.36 (17)
C4—C5—C6—N2	−177.2 (2)	O3—C13—C14—C15	−17.1 (2)
C2—C1—C6—C5	0.1 (3)	C12—C13—C14—C15	99.0 (2)
N1—C1—C6—C5	−177.46 (19)	O3—N3—C15—C16	−177.74 (17)
C2—C1—C6—N2	178.3 (2)	O3—N3—C15—C14	−1.2 (2)
N1—C1—C6—N2	0.7 (3)	C13—C14—C15—N3	12.0 (2)
C10—N2—C6—C5	−130.1 (2)	C13—C14—C15—C16	−171.60 (18)
C11—N2—C6—C5	41.1 (3)	N3—C15—C16—C21	165.85 (19)
C10—N2—C6—C1	51.7 (3)	C14—C15—C16—C21	−10.3 (3)
C11—N2—C6—C1	−137.1 (2)	N3—C15—C16—C17	−13.0 (3)
C1—N1—C8—O1	−176.2 (2)	C14—C15—C16—C17	170.9 (2)
C7—N1—C8—O1	−4.3 (3)	C21—C16—C17—C18	−1.7 (3)
C1—N1—C8—C9	4.7 (3)	C15—C16—C17—C18	177.11 (19)
C7—N1—C8—C9	176.56 (19)	C16—C17—C18—C19	−0.4 (3)
O1—C8—C9—C12	15.3 (3)	C17—C18—C19—C20	2.0 (3)
N1—C8—C9—C12	−165.63 (18)	C17—C18—C19—Cl1	−177.34 (16)
O1—C8—C9—C10	−107.9 (2)	C18—C19—C20—C21	−1.4 (3)
N1—C8—C9—C10	71.2 (2)	Cl1—C19—C20—C21	177.92 (15)
C6—N2—C10—O2	171.7 (2)	C19—C20—C21—C16	−0.8 (3)
C11—N2—C10—O2	0.3 (3)	C17—C16—C21—C20	2.3 (3)
C6—N2—C10—C9	−10.7 (3)	C15—C16—C21—C20	−176.55 (18)
